# COUGH: consolidating a mature field for the next 5 years

**DOI:** 10.1186/1745-9974-7-1

**Published:** 2011-04-10

**Authors:** Kian Fan Chung, Brendan Canning, Lorcan McGarvey

**Affiliations:** 1 National Heart & Lung Institute, Imperial College, London, UK; 2 John Hopkins Asthma Allergy Centre, Baltimore, MD, USA; 3 School of Medicine, Queen's University, Belfast, UK

## 

COUGH is now 5 years old and has been growing under the Editorship of Dr Rubaiyat Haque and Professor Fan Chung who have established it as a medium for cough work. The first Editorial of COUGH in 2005 entitled *COUGH: meeting the needs of a growing field *set out its objectives and we believe that COUGH has met the needs of a growing field. For the next 5 years, we will continue the path already laid out with a team of 3 co-editors (Brendan Canning, Fan Chung and Lorcan McGarvey), who will equally share the running of this journal.

One sign of the success of COUGH is the number of times that its top 10 published articles have been accessed on line over a one month period in June this year, which ranged from 221 to 669 times, attesting to the great visibility and interest on the web. All 10 articles were related to clinical aspects as well as to the clinical science of cough, and COUGH has published many frequently-accessed papers on basic mechanisms of cough. COUGH has particularly been the niche where the burgeoning field of development of ambulatory cough recordings has been publicised. Certainly in terms of quality, COUGH has achieved a high level.

Our aim during the next five years is to consolidate and expand the on-line Journal within the international medical and scientific publishing world with the overall vision of COUGH being the preferred medium for clinicians, clinical and basic scientists to publish their work on cough and cough-related areas. The optimism we have for these objectives rests on the increasing awareness of cough over the last 10 years as an area of unmet clinical needs. The pharmaceutical industry is very much interested in finding better treatments for cough. This is coupled with a greater interest in scientists in unravelling the cough receptors and its pathways.

The path for COUGH has been laid and established, and we will consolidate COUGH over the next 5 years. To do this, we plan to:

1. To increase further the international reach of COUGH. Cough is obviously a global problem, and there are important regional variations and specific challenges in cough. We would do this by expanding further the composition of the Editorial Board to include more representation from Asia, Africa and South America.

2. To work with expert groups that have an interest in cough within the Editorial Board including general practitioners, ENT, gastroenterology, cancer, and neurology experts, and scientists of related fields such as pain, breathlessness, and environmental science, including those working in industry.

3. To increase the publication of (i) basic scientific aspects of cough, with the aim of having at least 25% of the future publications (ii) articles relating to clinical/therapeutic aspects, with the realisation that there will be an increase in clinical trials of novel antitussive therapies in the next 5-10 years, as the methodology for cough trials is getting established and agreed upon

(iii) articles relating to the impact of cough in the common respiratory diseases particularly asthma, COPD and cancer.

4. To increase the number of *reviews *and to publish proceedings of cough and/or cough-related meetings, workshops, symposia and conferences, and to introduce new rubriques such as a *Year-in-Review *in subsections of cough field, and *News in Cough *to highlight important ongoing events/findings in the field.

We look forward to continuing support from the cough community with the submission of investigations and cough-related research to COUGH.

